# Coping profiles, depression, and body image anxiety during the Covid-19 pandemic: Comparative analysis of females with thyroid diseases and a non-clinical sample

**DOI:** 10.1371/journal.pone.0282302

**Published:** 2023-03-01

**Authors:** Marcin Rzeszutek, Małgorzata Pięta, Angelika Van Hoy, Magdalena Zawistowska, Monika Grymowicz, Wojciech Pięta, Sara Gołoś, Magdalena Walicka

**Affiliations:** 1 Faculty of Psychology, University of Warsaw, Stawki, Warsaw, Poland; 2 Department of Gynecological Endocrinology, Medical University of Warsaw, Warsaw, Poland; 3 Department of Internal Diseases, Endocrinology and Diabetology, Central Clinical Hospital of the Ministry of the Interior and Administration in Warsaw, Warsaw, Poland; 4 Department of Human Epigenetics, Mossakowski Medical Research Institute, Warsaw, Poland; The Hong Kong Polytechnic University, HONG KONG

## Abstract

**Objectives:**

This study aimed to compare profiles of coping among females with thyroid disorders and females from a healthy control group regarding depression levels and body image anxiety. We also wanted to check whether subjectively experienced Covid-19-related psychological distress moderated the above-mentioned association in both groups of participants.

**Method:**

The study sample comprised 564 females, of which 329 were diagnosed with a thyroid disease and 235 formed the healthy control group. Participants filled out paper-and-pencil or online versions of psychometric questionnaires to assess coping strategies, depression, and body image anxiety.

**Results:**

In general, we observed higher depression intensity and a higher level of body image anxiety among females with thyroid diseases than among the healthy control group. Latent profile analysis revealed adaptive vs. maladaptive coping profiles from both study samples. Depression symptoms were significantly higher if coping was maladaptive in both the clinical and control groups. Still, there were no significant differences in body image anxiety between participants with adaptive and maladaptive coping profiles. Covid-19-related distress did not moderate the link between coping profiles, depression, and body image anxiety in either group.

**Conclusion:**

Greater focus should be placed on the role of body image in females struggling with thyroid diseases. Bodily therapy may help these patients to cope better with co-occurring thyroid diseases and mental disorders, whose relationship is still not fully understood.

## Introduction

Thyroid diseases constitute a global health problem and are associated with substantial deterioration of physical and psychosocial quality of life among sufferers [see reviews and meta-analyses: 1,2]. Of the numerous thyroid disorders, autoimmune thyroid disease (AITD) is the most prevalent, ranging from Hashimoto’s thyroiditis, manifested by hypothyroidism or hyperthyroidism, to thyrotoxicosis caused by Graves’ disease [3,4]. The clinical presentation of thyroid diseases is highly variable and often nonspecific; consequently, the diagnosis of thyroid dysfunction is predominantly based on biochemical tests [5]. Hypothyroidism is a condition of thyroid hormone deficiency that leads to a generalized slowdown of metabolic processes [3]. As a result, the patient may develop various symptoms, such as weight gain, weakness, skin lesions, attention problems, and memory disorders. Hyperthyroidism is characterized by increased thyroid hormone synthesis and secretion from follicular cells [6]. Symptoms include weight loss, anxiety, irritability, insomnia, and, less frequently, psychotic manifestations. All thyroid disorders are much more commonly observed in females, although the reasons for this are not completely clear [3]. These frequent health problems lead many patients to remain under constant healthcare and strongly affect their psychological well-being. Epidemiological studies provide significant evidence of the associations between thyroid disorders and mental disorders [e.g., 1,2,7]. Our study focused on depression and anxiety related to perceived body image among females with thyroid diseases compared to females from a non-clinical sample.

Investigations of the link between thyroid dysfunction and psychopathology go back almost 200 years in medicine [8,9]. Several historical terms, such as *nervous affectations* and *myxedema madness*, have been used to underscore common-knowledge observations pointing to frequent mood swings, irritability, and worries among people suffering from thyroid diseases. Currently, the prevalence of mood and anxiety disorders in thyroid diseases accounts for up to 60% of anxiety disorders and even 70% of cases of depression [10,11]. Although the co-occurrence of these psychopathological disorders with thyroid disorders is well established, the mechanism of this association is still not fully understood [1,2]. Extensive community studies have found no significant impact of thyroid functions on depression in the general population [e.g., 12,13]. Attempts to directly link hormone imbalances or hormone treatment with psychopathological symptoms in patients suffering from thyroid diseases have also failed or provided conflicting results [see, e.g., 14–16]. Dayan and Panicker [17] underlined the problem of misattribution of thyroid dysfunctions with depressive or anxiety symptoms. That is, depression, anxiety, and thyroid diseases are relatively common medical conditions in similar populations, such as older women. For example, older women who are depressed may think it is because of a thyroid problem, even though there may be no connection. Several subjective cognitive factors are crucial in dealing with thyroid diseases [18]. In our study, we focused on subjective stress-coping strategies.

Although a considerable amount of research has provided solid evidence that coping matters concerning how people deal with stressful life events [see meta-analysis: 19], there is still no consensus on how to conceptualize, measure, and classify different ways of coping [see, e.g., 20–22]. One of the central problems in coping studies is that the term "coping" is often wrongly defined as an observable behavior or stable trait instead of being seen as a dynamic, multidimensional construct that encompasses various behaviors, emotions, and cognitions often used by the same person simultaneously [20,23,24]. Moreover, most authors still operationalize coping strategies using the variable-oriented approach, which describes the average associations between study variables for an entire sample [20]. Alternatively, in the variable-centered approach, the sample is treated as homogenous in terms of coping strategies, disregarding the fact that the same participant may have different positions on each coping dimension. In this context, the central research question is not why some coping strategies are less or more effective but rather how a specific person deals with a particular stressor and the extent of their effectiveness in doing so [25]. Therefore, in our study, we adopted a person-centered approach, focusing on the heterogeneity of the study participants in terms of coping profiles, which may be uniquely associated with the study variables [e.g., 26,27]. This latter approach has never been used in psychological studies of thyroid diseases. It thus may be beneficial in better understanding the vague mechanism linking psychopathological disorders in such patients [17].

The final variable of interest was subjective body image anxiety [28]. Traditionally, disturbances in body image have mainly been operationalized via the perceptual component of body image disturbance, that is, the overestimation of body size compared to the current societal norms [see, e.g., 29–31]. However, nowadays, disturbed body image is associated not only with body weight but also with non-weight factors [e.g., ears, lips, hands, and feet; 32,33]. In addition, the perfection of physical appearance is increasingly challenging to achieve, and an increasing number of people experience anxiety related to their "imperfect body image" [28,34]. Body image anxiety was defined as anxiety on the cognitive, affective, and perceptual levels, manifested by a person’s dissatisfaction with their physical appearance and having two distinct components: state and trait [28]. Whereas body-state anxiety is transient emotional distress that depends on the current situational context, body-trait anxiety is a relatively stable misperception of one’s body, probably rooted in personality factors [35]. As mentioned above, thyroid disorders manifest with versatile symptoms related to negative body image [1,2]. Thus, in our study, we wanted to compare the state versus trait body image anxiety among females with thyroid disorders and females in a non-clinical sample in the critical situational context, namely, the Covid-19 pandemic.

## Method

### Study aims

**Fig 1:**
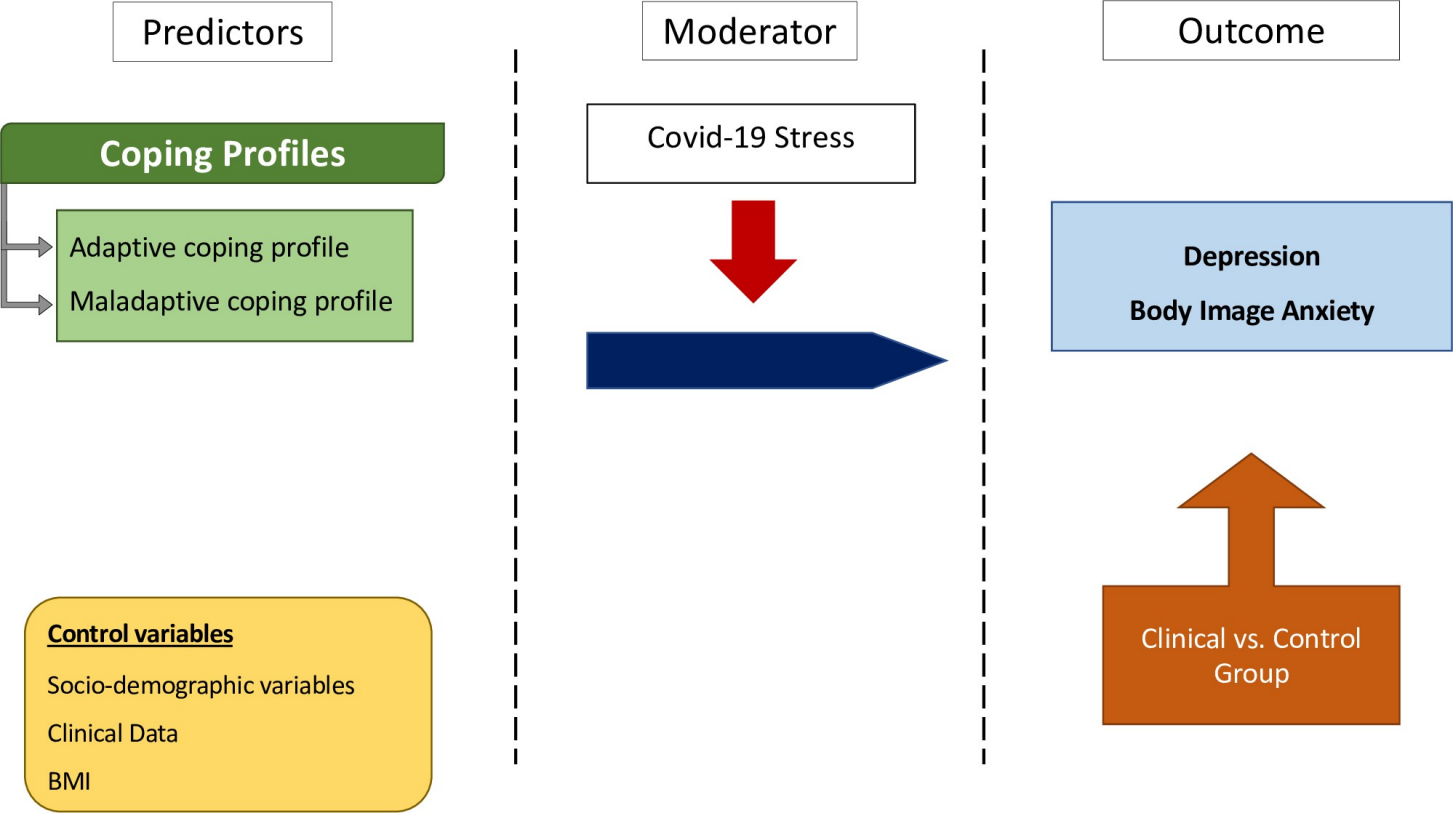
Data analysis plan.

The study had two aims. First, we wanted to determine whether we could observe different profiles of coping among females with thyroid disorders and females from the healthy control group concerning depression level and body image anxiety. Second, we wanted to check whether both samples’ subjectively experienced Covid-19-related psychological distress moderated the relationship between coping profiles, depression, and body image anxiety. To the best of our knowledge, there are no studies conducted with females with thyroid disorders that might be useful as a direct source of research hypotheses in relation to this unique study design, particularly with a control group. Therefore, we mainly employed an exploratory approach in our study. However, based on existing studies within a similar methodological framework but conducted on different samples of participants [see 26,27], we formulated two hypotheses, and our analytical plan is shown in Fig 1.

*Hypothesis 1*. We expected that, in general, females with thyroid diseases would score higher on depression and state and trait body image anxiety compared to females in the control group.

However, the picture of these differences may be modified if coping is included in our analysis. More specifically, we hypothesize that adaptive and maladaptive coping profiles (see Measures section) may be found among the participants. Adaptive coping profiles will be negatively associated with the intensity of depression and body image anxiety (on both the state and trait levels). The opposite trend will be observed in maladaptive coping profiles.

*Hypothesis 2*. We expected that the aforementioned relationships between coping profiles, depression, and body image anxiety would be moderated by subjectively experienced Covid-19-related psychological distress.

### Data analysis

Firstly, the characteristics of the sample in the current study were calculated in order to verify if the two main groups differ and how it limits the conclusions. Next, distributions of analysed interval variables were assessed with the use of measures for skewness and kurtosis. The clinical group and the control group were compared in terms of body image anxiety and depression with the use of independent samples t-test. Then, Latent Profile Analysis was conducted to extract distinct profiles of coping. The participants with extracted coping profiles were compared in terms of body image anxiety and depression separately in the clinical group and the control group also with the use of independent samples t-test. To assess if Covid-19 distress was a moderator of the relationships between coping style and body image anxiety and depression a hierarchical regression analysis with focus on interaction effects was applied.

### Participants and procedure

This was a cross-sectional, comparative study design with two study groups: clinical and control. Female participants from the clinical group, i.e. with thyroid disorders were recruited from the Department of Gynecological Endocrinology at the Medical University of Warsaw and the Endocrinology Clinic of the Central Clinical Hospital of the Ministry of the Interior and Administration in Warsaw. The participants from the clinical sample completed paper-and-pencil questionnaires. The control group was recruited via social media platforms and completed the study measures online. In this study, we included a control group as we wanted to compare the way of coping with stress in health and disease during the Covid-19 pandemic with a particular focus on depression and body image anxiety in this matter. For the clinical sample, the eligibility criteria were being 18 years or older and having a medical diagnosis of thyroid disorder, which was screened by medical doctors working in the hospitals where the research was conducted. The inclusion criteria for the control sample were 18 years or older and declaring no chronic illness. The study occurred between September 2021 and February 2022; a time described as the "fourth wave" of the Covid-19 pandemic in Poland.

All procedures performed in this study were per the ethical standards of the institutional research committee and with the 1964 Helsinki Declaration and its later amendments. All groups voluntarily participated in the study and written informed consent was obtained from all the study participants. The local ethics committee approved our study.

### Measures

#### Coping

Stress-coping strategies were evaluated using Carver’s Brief COPE inventory [36] adapted for Polish. This inventory measures dispositional stress coping operationalized as the typical pattern of a particular person’s reactions and feelings under high stress. The inventory consists of 28 items, which form 14 subscales describing several stress-coping strategies, including usually adaptive patterns of coping [see: 22]–problem-focused coping (active coping, planning, use of instrumental support), emotion-focused coping (use of emotional support, positive emotion enhancement), and meaning-focused coping strategies (positive reframing, humor, acceptance, religion)—and usually maladaptive, avoidance coping strategies (self-distraction, denial, substance use, behavioral disengagement, venting, self-blame, rumination). Participants rated the frequency of particular coping behaviors on a 4-point Likert scale ranging from 0 (“Completely disagree”) to 3 (“Completely agree”) in response to individual sentences from the inventory.

#### Depression

We used the Centre for Epidemiological Studies Depression Scale [CES-D; 37] adapted for Polish. The CES-D is an internationally recognized questionnaire to assess the level of depression in clinical trials and epidemiological studies. Participants were asked on a 5-point Likert scale ranging from 1 ("Rarely") to 4 ("Almost always") how often they had felt particular depressive symptoms in the last week. The higher the score on the CES-D, the higher the depression level.

#### Body image anxiety

We used the state and trait versions of the Physical Appearance State and Trait Anxiety Scale [PASTAS; 28] adapted for Polish. The PASTAS is a questionnaire used to evaluate how anxious, tense, or nervous participants feel in the current situation (see state version) and generally (i.e., usually; see trait version) about specific body parts. The PASTAS is composed of 16 items belonging to two dimensions, one related to body weight (8 items; e.g., “My stomach”) and one to body areas not related to body weight (8 items; e.g., “My lips”). Participants respond using a 5-point Likert scale ranging from 0 (“Never”) to 4 (“Almost always") for anxiety about particular parts of their body. In our study, in the state version, we instructed both samples to focus mainly on body image anxiety experienced during the crisis period, that is, the Covid-19 pandemic.

#### Covid-19 distress

We used a short but reliable operationalization of Covid-19 distress based on other studies published prior to the commencement of our research [e.g., 38]. We asked participants on a 5-point Likert scale how stressful (in general) the pandemic and their life during it had been. The answers ranged from 1 (“Not at all)” to 5 (“Very much”).

## Results

Participants in the study comprised 564 women aged 18–82 (M = 38.32; SD = 17.54), 329 of whom were diagnosed with thyroid diseases (58.3%) and 235 of whom formed the control group (41.7%). Table 1 presents the study participants’ sociodemographic and (in the case of females with thyroid disorders) clinical characteristics.

**Table 1:** Demographic characteristics of entire sample (n = 564).

		Control Group		Clinical Group	
		n	%	n	%
Age		18–66	M = 26.83; SD = 10.41	18–82	M = 46.55; SD = 16.92
Relationship	In stable relationship	143	60.9	243	73.9
Education	Primary	1	.4	7	2.1
	Vocational	1	.4	15	4.6
	Secondary	181	77.0	108	32.8
	Higher	52	22.1	199	60.5
Employment	Regular employment	169	71.9	203	61.7
	Unemployed	62	26.4	34	10.3
	Pension	0	0	3	.9
	Retired	4	1.7	89	27.1
Financial	Very good	15	6.4	30	9.1
status	Good	100	42.6	166	50.5
	Medium	112	47.7	120	36.5
	Bad	6	2.6	12	3.6
	Very bad	2	.9	1	0.3
Place	City > 100,000 inhabitants	98	41.7	193	58.7
of residence	City > 50–100,000 inhabitants	25	10.6	42	12.8
	City < 50,000 inhabitants	62	26.4	48	14.6
	Village	50	21.3	46	14.0
Diagnosis	Hyperthyroidism	-	-	35	14.9
	Hypothyroidism	-	-	158	67.2
	Hashimoto’s disease	-	-	151	64.3
	Other	-	-	74	31.5
Symptoms	Dry skin	-	-	194	82.6
	Tiredness	-	-	210	89.4
	Memory impairment	-	-	125	53.2
	Weight gain	-	-	161	68.5
	Constipation/Diarrhea	-	-	79	33.6
	Feeling cold	-	-	117	49.8
	Swelling	-	-	63	26.8
	Feeling hot	-	-	56	23.8
	Weight loss	-	-	10	4.3
	Fast heartbeat	-	-	89	37.9
	Nervousness	-	-	160	68.1
Clinical depression	CES-D total score > 16	10	4.2	20	6.1

Most participants in both groups were in a stable relationship, had a secondary or higher education, and were regularly employed. Their financial status was medium or good. Most lived in cities with over 100,000 inhabitants. Most of those in the clinical group were diagnosed with hypothyroidism. The treatment period ranged from just diagnosed to 52 years (M = 10.70; SD = 9.24). Most participants from the clinical group experienced dry skin, tiredness, weight gain, and nervousness.

Table 2 presents the descriptive statistics for the analyzed variables: the mean values, standard deviations, minimum and maximum values, and the values for skewness and kurtosis. The skewness and kurtosis values did not exceed the range from -1.0 to 1.0. Therefore, parametric statistical tests were used in the subsequent analysis.

**Table 2:** Descriptive statistics for analyzed variables.

Variables	M	SD	min	max	S	K	α
BMI	24.89	5.92	12.69	48.79	.21	.38	-
Active coping	3.81	1.34	0	6	-.46	.35	.65
Planning	3.89	1.34	0	6	-.64	.71	.67
Positive reframing	3.35	1.71	0	24	.82	.56	.51
Acceptance	3.78	1.26	0	6	-.68	.16	.48
Humor	2.08	1.28	0	6	.17	-.23	.64
Religion	1.90	1.93	0	6	.54	-.98	.86
Use of emotional support	3.07	1.27	0	6	-.18	.01	.62
Use of instrumental support	3.34	1.62	0	6	-.41	-.49	.67
Self-distraction	3.62	1.47	0	6	-.44	-.10	.64
Denial	1.84	1.60	0	6	.56	-.49	.64
Venting	3.09	1.46	0	6	-.25	-.13	.63
Substance use	0.97	1.50	0	6	.52	.44	.87
Behavioral disengagement	1.67	1.43	0	6	.71	.22	.64
Self-blame	2.48	1.77	0	6	.25	-.86	.72
Depression	36.11	12.44	2	73	.58	-.19	.91
State body image anxiety	15.43	12.33	0	64	.68	.14	.91
Trait body image anxiety	15.85	14.02	0	64	.88	.33	.94
Covid distress	3.35	1.09	1	5	-.25	-.57	-

**Legend:** M = mean value; SD = standard deviation; min = minimum value; max = maximum value; S = skewness; K = kurtosis; α = Cronbach’s α reliability coefficient.

To start verifying Hypothesis 1, participants from the control group were compared in terms of body image anxiety and depression separately from participants in the clinical group. The results are presented in Table 3.

**Table 3:** Mean values of body image anxiety and depression for both groups.

	Group						
	Control		Clinical				
	M	SD	M	SD	*t*	df	p
State body image anxiety	14.01	12.88	16.44	11.84	-2.32	562	.021
Trait body image anxiety	13.65	13.96	17.43	13.87	-3.18	562	.002
Depression	35.33	11.34	37.21	13.79	4.27	547.58	.001

**Legend:** M = mean value; SD = standard deviation; *t* = value of independent samples; *t-*test; df = degrees of freedom; p = statistical significance.

The intensity of body image anxiety, both as a state and as a trait, and the level of depression were significantly higher in the clinical group.

**Fig 2:**
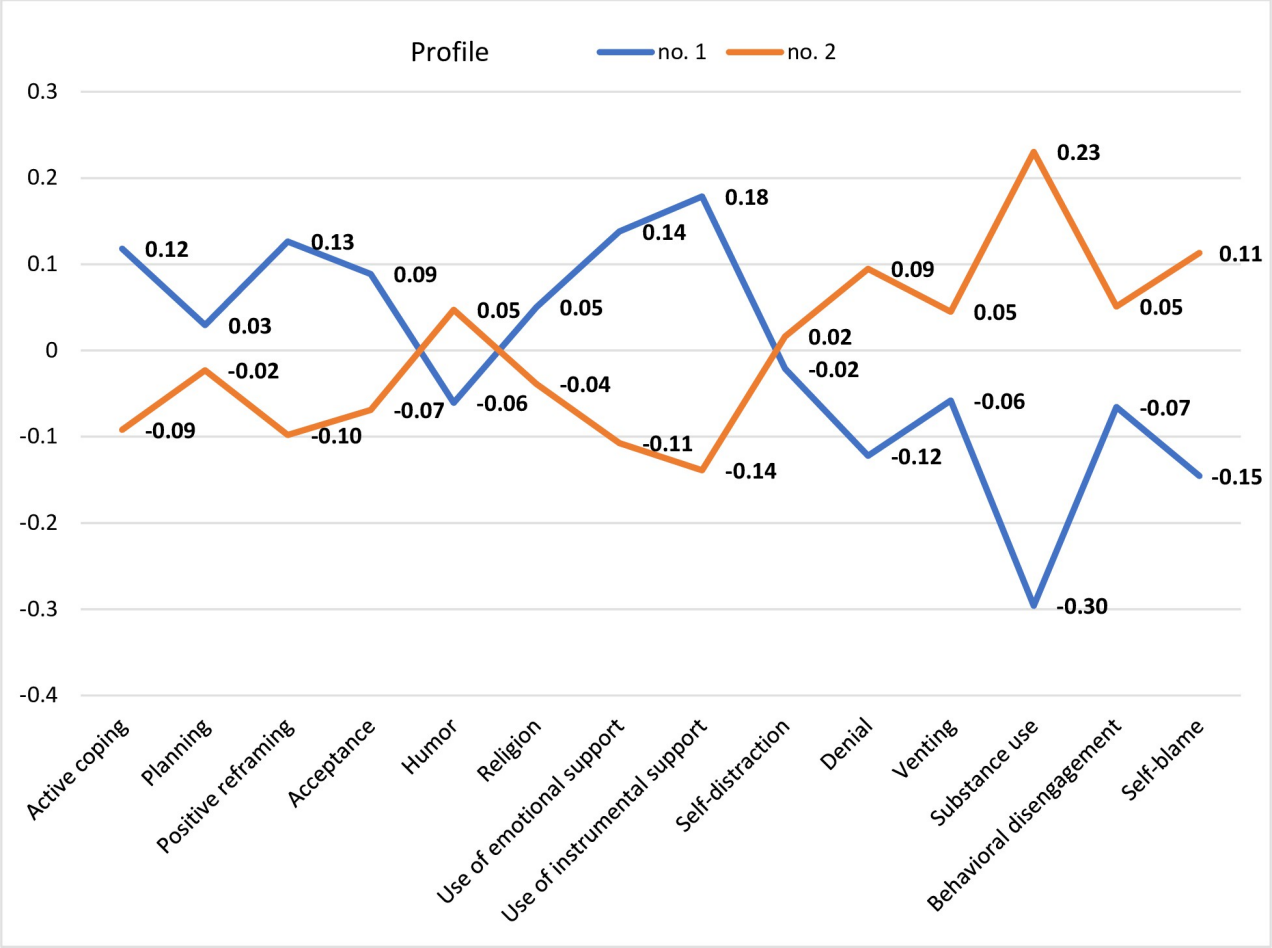
Profiles of coping extracted in the studied samples.

The main analysis was based on a Latent Profile Analysis [LPA; 39]. This was conducted to extract distinct profiles of coping. According to the values of the Aikake information criterion (AIC) and Bayesian information criterion (BIC), the model with best fit was the model with varying variances and varying covariances and with two extracted classes with two distinctive profiles. The values of fit statistics were AIC = 20,349.06 and BIC = 21,385,14. Fig 2 presents the adaptive and maladaptive coping profiles extracted from the study participants.

The extracted classes included a group of participants with adaptive coping (*Profile 1*) and a group with maladaptive coping (*Profile 2*). Adaptive coping was more prevalent in the clinical group. It was present in 181 participants in the clinical group (55.0%) and 78 participants in the control group (33.2%). According to the Pearson’s chi-square test of independence, the relationship between group membership and coping style profile was statistically significant, χ^2^(1) = 26.29, *p* < .001.

To verify Hypothesis 1, participants with the extracted adaptive coping profile (*Profile 1*) and participants with the maladaptive coping profile (*Profile 2*) were separately compared in terms of body image anxiety and depression in the clinical group and the control group. The results are presented in Table 4.

**Table 4:** Mean values of body image anxiety and depression in group with adaptive coping and group with maladaptive coping.

		Coping Profiles						
		Adaptive		Maladaptive				
Group		M	SD	M	SD	*t*	df	p
Control group	State Body Image Anxiety	12.03	9.52	14.99	14.19	-1.90	212.78	.059
	Trait Body Image Anxiety	11.31	11.13	14.82	15.07	-2.01	199.54	.045
	Depression	34.41	10.47	38.61	15.01	-2.49	207.62	.014
Clinical group	State Body Image Anxiety	15.56	11.14	17.52	12.61	-1.48	296.02	.141
	Trait Body Image Anxiety	16.70	13.59	18.32	14.20	-1.05	308.39	.296
	Depression	33.51	9.80	37.55	12.66	-3.18	272.78	.002

**Legend:** M = mean value; SD = standard deviation; *t* = value of independent samples *t-*test; df = degrees of freedom; p = statistical significance.

For the mean values of body image anxiety, both state and trait were higher when coping was maladaptive in the control group. However, there were no significant differences in body image anxiety between participants with adaptive coping and participants with maladaptive coping in the clinical group. Depression symptoms were significantly higher if coping was maladaptive in both the clinical and control groups. In the control group, BMI was positively related to body image anxiety for both states, *r*(233) = .22, *p* < .001, and trait, *r*(233) = .16, *p* < .001, but it was not significantly related to body image anxiety in the clinical group for either state, *r*(326) = .04, *p*>.05, or trait, *r*(326) = .02, *p*>.05. Finally, BMI did not correlate with depression in the clinical group, *r*(326) = .03, *p*>.05, or in the control group, *r*(233) = -.01, *p*>.05.

To verify Hypothesis 2, a hierarchical regression analysis was carried out. The interaction effects between coping style and the Covid-19 level of stress intensity were used as indicators for moderation. First, to provide context for the interpretation of potential interaction effects we compared the mean level of Covid-19 psychological distress between the two groups of participants. The mean level of Covid-19 psychological distress in the clinical group was 3.54, with a standard deviation of 1.06. The mean level of Covid-19 psychological distress in the control group was 3.10, with a standard deviation of 1.08. According to the value of an independent samples *t-*test, the difference was statistically significant, *t*(562) = -4.74, *p* < .001, meaning that the level of Covid-19 distress was significantly higher in the clinical group. Second, we conducted a regression analysis separately for the clinical and control groups. The interaction effects between coping style and the Covid-19 level of stress intensity were not statistically significant. This applied to models in which the explained variable was body image anxiety as a state in the control group, *B* = -.03, *t* = -.42, *p*>.05, body image anxiety as a trait in the control group, *B* = -.08, *t* = -1.14, *p*>.05, depression in the control group, *B* = -.03, *t* = -.45, *p*>.05, body image anxiety as a state in the clinical group, *B* = .01, *t* = .22, *p*>.05, body image anxiety as a trait in the clinical group, *B* = .04, *t* = .76, *p*>.05, and depression in the clinical group, *B* = -.01, *t* = -.09, *p*>.05. Therefore, no moderation effects were detected.

## Discussion

The results of our study were consistent with our first hypothesis. We observed higher depression intensity and a higher level of body image anxiety (in both state and trait dimensions) among females with thyroid diseases than among the healthy control group. We also extracted adaptive vs. maladaptive coping profiles from both study samples, as shown in Fig 2. Interestingly, the adaptive coping profile was significantly more prevalent in the group of women with thyroid diseases, but it did not have any relationship with body image anxiety. Moreover, in females with thyroid disorders, body image anxiety was not linked to BMI. Opposite trends were observed in the healthy control group; among these females, body image anxiety was related to coping and BMI.

Our findings have important implications for the psychological functioning of females with thyroid dysfunction as operationalized by body image. Several authors have observed relatively impaired psychosocial well-being and poor quality of life among females with thyroid disorders compared to healthy controls [40,41]. Nevertheless, until now, body image among females with thyroid dysfunctions has been studied predominantly among patients after thyroid surgery [e.g., 42,43], and thus little is known about how these women experience their body as a part of their everyday quality of life. Although they try to adapt to their illness and use various coping strategies, subjective (coping) and objective (BMI) factors do not matter for their overall highly negative view of their body and related anxiety. Perhaps these physical factors should be considered when trying to understand the still-unknown mechanism of the co-occurrence of psychopathological disorders in thyroid disorders and when proposing the direction of therapy [1,2]. Some authors recommend including somatic treatment in health care programs (e.g., yoga practice); such therapy has been found to reduce illness-related distress and enhance the emotional aspects of quality of life in those with thyroid disorders [44]. In addition, to better understand the processes underlying negative experiences of the body in thyroid disease, one may underline the constant somatic threat, as described in the *enduring somatic threat model of posttraumatic stress disorder* of Edmondson [45]. Although thyroid diseases are not life-threatening, they can still increase alertness and alienate patients from their bodies, which they may consider a source of constant threat and stigma [41].

Concerning depression, maladaptive coping was a significant predictor of depressive symptoms in both groups. Still, as with body image anxiety, depression was not linked to BMI in either group. Engum et al. [46] argued that while the causal relationship between thyroid diseases and anxiety and depression disorders remains unclear, these psychiatric disorders should be diagnosed and treated separately because depression and anxiety may be able to coexist in this patient group by chance, without any impact on each other. In addition, other studies have shown that the level of emotional distress in females struggling with thyroid dysfunctions is not related to clinical markers (thyroid hormones) or even medical treatment [47]. More recently, Pankowski et al. [18] highlighted the role of negative mental illness beliefs in females with thyroid disorders; these beliefs were shown to be the strongest predictor of emotional distress regardless of sociodemographic factors, hormone levels, or even time since diagnosis. These negative cognitions are mainly related to personal shame and stigma about the visibility of symptoms and anticipation of negative social attitudes toward them. Thus, it seems that psychosocial interventions to reduce depression among thyroid patients should focus on reducing negative shame-related beliefs by promoting positive stress-coping strategies. This approach effectively reduces depressive symptoms for other chronic illnesses [27,48].

Our second hypothesis was not confirmed. The intensity of Covid-19-related distress did not moderate the relationship between coping profiles, depression, and body image anxiety either in the clinical sample or in the control group (see: Data analysis plan, Fig 1). However, Covid-19 psychological distress was higher in the clinical group of females. This rather unexpected finding can be interpreted in two ways. First, it seems that among females with thyroid disorders, the stress associated with the pandemic did not affect their overall negative emotional functioning, as operationalized in terms of their body image anxiety or depressive symptoms. In other words, it may be that their illness-related distress was mostly an internally induced permanent state of mind, resistant to objective illness progression, medical treatment, and even extraordinary external circumstances [27,46]. On the other hand, our findings can be interpreted in a broader context. The initial period of the Covid-19 pandemic saw much debate over its impact on the level of mental health problems in the general population. However, recent extensive epidemiological studies have shown that these catastrophic projections were highly exaggerated, and many people even reported some positive changes due to the new lifestyle imposed by the pandemic [49]. This corresponds particularly with research on the quality of life among pregnant women during the Covid-19 pandemic, whose emotional well-being was even better than before the pandemic due to the greater emotional support received at this critical time, as well as the more subjective sense of the closeness of family life [50,51]. Although this topic requires further study, this may explain why the Covid-19 pandemic did not have any association with body image anxiety or depression in the control group.

### Strengths and limitations

This study has several strengths, including its large clinical sample with a healthy control group observed in the critical moment of the Covid-19 pandemic, its innovative methodological design (person-centered approach), and its focus on an understudied variable in this illness, namely, body image anxiety. However, we should also note several limitations. First, due to organizational reasons, our clinical sample was heterogeneous in terms of thyroid disease diagnoses and stages of the disease. We also controlled for a relatively low number of clinical markers because the main focus was on psychological functioning. Second, because of organizational difficulties, our control group of healthy females was not entirely similar in terms of their various sociodemographic factors. Third, the cross-sectional design of this study precludes causal interpretations of the observed findings.

## Conclusion

Despite these limitations, our study addresses significant research gaps in the literature on psychological functioning among females struggling with thyroid diseases. It indicates, in particular, that a greater focus is required in healthcare programs for these patients on their body image and related dysfunctional emotions and cognitions. Perhaps bodily therapy could help these patients to cope better with mental disorders co-occurring with thyroid diseases, whose relationship is still not fully understood. More specifically, various programs have been created over several years to shape a positive body image, creating analogous interventions focusing on clients’ strengths and resources in different clinical and non-clinical samples [52]. Concerning the high degree of subjective shame and personal stigma experienced by females with thyroid diseases, the shift from simply reducing negative body image to promoting its positive aspects may be a promising direction for psychosocial interventions devoted to these patients.

## Supporting information

S1 Dataset (XLSX)
